# Carer quality of life and experiences of health services: a cross-sectional survey across three neurological conditions

**DOI:** 10.1186/1477-7525-11-103

**Published:** 2013-06-25

**Authors:** Michele Peters, Crispin Jenkinson, Helen Doll, E Diane Playford, Ray Fitzpatrick

**Affiliations:** 1 Department of Public Health, University of Oxford, Old Road Campus, Oxford OX3 7LF, UK; 2 Oxford Outcomes, Seacourt Tower, West Way, Oxford OX2 0JJ, UK; 3 University College London, Institute of Neurology, Queen Square, London W1N 3BG, UK

**Keywords:** Carer, Quality of life, Experiences, Health care, Neurology

## Abstract

**Background:**

Neurological conditions have a substantial impact on carers, with carer well-being having been shown to be influenced by a number of demographic, patient and caregiving factors. Support given to carers can lead to better coping. This study investigated the relationship between carer well-being and experiences with health and social services.

**Methods:**

A cross-sectional survey was conducted of 1910 (37.4%) of carers of 5109 people with motor neuron disease (MND) (n=434, 54.9%), multiple sclerosis (MS) (n=721, 30.7%) and Parkinson’s disease (PD) (n=755, 38.2%). Carers completed a generic health status measure (SF-12), a carer strain measure (Carer Strain Index- CSI) and a newly developed questionnaire on health and social care experiences. Data were analysed by analysis of variance with p set at <0.05.

**Results:**

Carer well-being was found to be compromised and differed significantly between the three conditions. Furthermore, a considerable number of carers experienced problems with aspects of health and social care, although there was no clear pattern according to the condition that was cared for. The total number of problems reported did not differ significantly between conditions but was significantly (all p<0.001) associated with carer quality of life (both physical and mental health) and strain, even when other influencing factors (demographic and caregiving variables) were corrected for. The association was particularly strong for carer strain, and less strong (but still significant) for quality of life.

**Conclusions:**

The results show that carer well-being is compromised, in line with previous studies. Furthermore, the link of carer well-being to the number of problems reported suggests that minimizing problems experienced could improve carer well-being. This stresses the importance of health and social services appropriately supporting carers.

## Background

Motor neuron disease (MND), multiple sclerosis (MS) and Parkinson’s disease (PD) are three long-term neurological conditions (LTNCs) that affect the quality of life of people with these conditions [1-4]. These LTNCs are progressive meaning that disability increases over time, with support from others, usually family members, being needed and tending to increase over time as symptoms become more severe [5]. As informal caregivers play an important role in the care for LTNCs, it is important to consider their well-being and the support that health and social services can provide to help carers in the caregiving role. Quality of life of caregivers of someone with one of these three LTNCs is impaired [2,4,6-8], and quality of life is poorer when the symptoms are more severe, the amount of time spent caring increases or if the patient is depressed [4,5,7,9-11].

Caring for someone with an LTNC can constitute a considerable burden. MND caregivers reported that the main burdens were ‘personal and social restrictions’ and ‘physical and emotional problems’ [12]. In MS carers the main health problems are anxiety, depression and tiredness [5] and a systematic review concluded that providing care in MS has a major impact on all areas of the caregiver’s life [13]. Family and friends spent 4.6 to 12 hours per day providing care to moderately and severely disabled MS patients [14]. In PD, caregivers have more severe depression and higher reports of tiredness, sadness and lower life satisfaction than healthy elderly people [15], and experience considerable burden in terms of health, depression and social life [5], psychological distress [16] and strain from their caregiving role [17]. PD caregivers also report tasks involving physical effort to be the most difficult [18].

Due to the impact on caregivers, it is important that appropriate support is provided with the goal of minimising the impact on quality of life and reducing caregiver related burden. Although many complexities surround caregivers’ decisions to seek and accept support [19], perceived social support in MS has been shown to be beneficial to the caregiver [13]. Although this was not support specifically from health services, it is reasonable to assume that such support would benefit caregivers. Generally, little information is available on the impact of support of health and social care services on caregiver well-being. However, a study of people with a neurodegenerative condition (including MND, MS, PD and Huntington’s Disease) and their caregivers showed that those receiving more tailored health services and more palliative care services were more satisfied with their care [20]. Furthermore, MND caregivers’ satisfaction with formal services correlates with caregivers’ self-predicted ability to cope in the future [21]. This study aims to examine the relationship between quality of life in LTNC caregivers and support provided by health and social care services, with a particular focus on problems experienced with support from services.

## Methods

A cross-sectional postal survey was conducted to gather information on people with a long-term neurological condition and their caregiver’s health status and experiences of health and social care services (October 2008 to January 2009). This article presents the findings on caregivers’ health status and experiences of services.

Ethical approval for the survey was obtained through the Central University Research Ethics Committee (University of Oxford).

### Sample

Individuals with MND, MS and PD were recruited through the MND Association, Parkinson’s UK (formerly the PD Society) and the MS Society and were asked to give the caregiver questionnaire to their main informal caregiver, if they had a one. In this study, ‘caregiver’ was defined as ‘a family member or friend who provides unpaid care (such as help with dressing and feeding or help with housework) to the patient’.

The survey was sent to 5109 (790 MND, 2345 MS and 1974 PD) members of the respective patient organisations. The majority were postal questionnaires, but 190 people with MND were sent an electronic version of the survey using SurveyMonkey (http://www.surveymonkey.com). All surveys were sent by the patient organisations for reasons of data protection. A ‘thank you’ note/reminder was sent two weeks after the initial mail out of the questionnaires or email invitation.

### Questionnaires

Caregivers completed a generic health status measure, a carer specific measure, and additional questions on experiences of health and social care services, as well as standard demographic questions.

### Health status questionnaires

The generic measure was the 12-item Short Form Health Survey (SF-12v2), which is derived from its ‘parent measure’ the 36-item Short Form Health Survey (SF-36) [22,23], with the 12 items reproducing 90% of the variance in the overall Physical and Mental Health components of the 36 item parent form [24]. The SF-12v2 asks respondents about aspects of their daily lives over the past four weeks [25] and produces two summary scores - the Physical Component Summary (PCS) and the Mental Component Summary (MCS). The PCS and MCS are generated using norm-based methods and are standardised, using scores from the general working age population, to have a mean of 50 (S.D.10). A higher score indicates better quality of life.

The Caregiver Strain Index (CSI) was selected as the carer-specific measure [26]. The CSI asks whether aspects of the caregivers’ lives, such as sleep, finances and normal routine have been affected by their caring role, and whether this has placed a physical and mental strain upon them. The version of the CSI used in this study includes the original thirteen items but with an amended response set with three response options to each item (‘never’, ‘sometimes’, ‘always’). A higher CSI score means a higher burden (with scores calculated in the range 0–26).

### Experiences of health and social care services questionnaires

A set of items was developed to assess caregiver experiences of health and social care services in relation to their caring responsibilities. Item development was an iterative process based on a literature review and caregiver input. Twelve caregivers contributed to questionnaire development through qualitative interviews and 3 caregivers gave feedback throughout the developmental phase. Six caregivers participated in cognitive interviews which tested comprehension. The questionnaire was redrafted based on their comments before conducting a pilot study. A total of 212 carers (105 MND, 38 MS and 69 PD) participated in the pilot (overall response rate 43.8%). The pilot data were analysed for missing data. Any comments given by respondents were summarised to identify any commonly reported problems. The questionnaire was redrafted to produce the final version which covers aspects caregiver experiences. The experience items included in the analysis relate to perceived help and support provided to caregivers by health and social care professionals.

### Analysis

All questionnaires were double entered and verified. Discrepancies between the first and second sets of data entry were corrected before data analysis using SPSS v18.0.

The method of analyses adopted for the caregivers’ experience questions is similar to that adopted for Picker Patient Survey questionnaires [27,28]. Each item is re-coded as a dichotomous ‘problem score’ , indicating the presence or absence of a problem. A problem is defined as an aspect of health care that could, in the eyes of the caregiver, be improved upon. Chi-squared tests were used to identify significant differences between the LTNCs in the number of carers who reported a problem with each of the 12 items of health and social care. Subsequently, the items were summed to derive a total problem score on the basis of the items’ face validity and internal consistency reliability. As there were 12 items, the total problem score ranged from ‘0’ (i.e. no problems reported) to ‘12’ (i.e. a problem reported with each item). As the total problem score was not normally distributed, it was transformed to a categorical variable (0=0 problems, 1= 1 problem, 2=2-3 problems, 3 = 4–5 problems and 4= 6 or more problems).

Mean PCS, MCS and CSI scores were calculated for carers of each of the three LTNCs. Analysis of variance (ANOVA) was used to identify significant relationships between type of LTNC, demographic and caring variables, and the total problem score with PCS, MCS and CSI score. Finally, an analysis of covariance (ANCOVA) was conducted to examine the association between total problem score and PCS, MCS and CSI scores adjusting for the type of LTNC, and with demographic and caring variables as covariates. No data imputation methods were used to calculate the PCS, MCS, CSI or problem items, therefore it was not always possible to calculate dimension and/or total problem scores for every respondent. The level of significance was set at p<0.05 for all analyses.

## Results

### Demographics and caring role

A total of 1910 caregivers (37.4% of the 5109 patients contacted) participated in the survey, including 434 MND (54.9% of 790 patients), 721 MS (30.7% of 2345) and 755 PD (38.2% of 1974) caregivers, with an overall mean (SD) age of 62.83 (12.16) years [MND 62.98 (11.98), MS 58.01 (12.09) and PD 67.32 (10.48); p<0.001]. The majority of carers were female (n=1096, 57.8%), although MS carers were more likely to be male (n=453, 63.4%; p<0.001). The length of time in the caregiving role was longer for MS and PD (p<0.001) but fewer hours were spent on caregiving tasks than in MND (p<0.001). Full details of these data are given in Table 1.

**Table 1:** Demographics and caring role

		**MND**		**MS**		**PD**		**p**
		**N**	**%**	**N**	**%**	**N**	**%**	
**Gender**	*Male*	134	31.2	453	63.4	213	28.3	<0.001
	*Female*	296	68.8	261	36.6	539	71.7	
**Time as a carer**	*< 1 year*	90	21.0	20	2.8	20	2.7	<0.001
	*1-2 years*	173	40.3	55	7.7	94	12.6	
	*3-4 years*	70	16.3	70	9.8	179	23.9	
	*5-10 years*	61	14.2	220	30.7	250	33.4	
	*>10 years*	35	8.2	352	49.1	205	27.4	
**Hours spent caring/ week**	*1-19*	99	23.2	256	36.4	245	34.1	<0.001
	*20-34*	80	18.7	123	17.5	115	16.0	
	*35-70*	92	21.5	156	22.2	162	22.5	
	*>70*	156	36.5	169	24.0	197	27.4	
		Mean	SD	Mean	SD	Mean	SD	
**Age**		62.98	11.98	58.01	12.09	67.32	10.48	<0.001

### Carer quality of life and strain

Carer quality of life across all three conditions, assessed by the SF-12, was lower than the population norm for both physical (PCS) and mental (MCS) health. The mean (SD) overall PCS score was 47.64 (11.56) and MCS 44.62 (11.15). Significant differences were found between the three LTNCs, with PD carers reporting the lowest physical health score and MND carers reporting the lowest mental health score (both p<0.001) (Table 2). The CSI score was highest in MND carers, indicating that MND carers experience a higher strain from their caregiving role (p<0.001) (Table 2).

**Table 2:** Carer quality of life and strain

		**N**	**Mean**	**95% CI**	**p**
**SF-12 physical health**	MND	363	48.04	46.83 - 49.25	<0.001
	MS	641	48.79	47.93 - 49.65	
	PD	626	46.24	45.31 - 47.16	
**SF-12 mental health**	MND	363	43.01	41.82 - 44.21	<0.001
	MS	641	46.12	45.28 - 46.96	
	PD	626	44.02	43.16 - 44.89	
**Carer strain index**	MND	362	14.69	14.01 - 15.38	<0.001
	MS	633	11.44	10.93 - 11.95	
	PD	571	11.89	11.36 - 12.41	

### Carer experiences of services

Twelve different areas of health and social care services had been examined and in half of these significant differences were found between the LTNCs (Table 3). In some domains significantly more MND carers experienced problems, whereas in others they experienced significantly fewer. For most areas, the proportion of carers reporting problems were similar in MS and PD, except ‘receiving financial support’ where a problem was less frequently reported by PD carers. The total problem score was significantly different by LTNC (p<0.001) (Table 4).

**Table 3:** Carer experiences of health and social care

	**MND**	**MS**	**PD**	
**Items**	**n (%)**	**n (%)**	**n (%)**	**p**
Did not have a carer assessment but would have liked one	113 (26.5)	158 (22.2)	154 (20.8)	NS
No/ not enough opportunity to discuss amount of caring with a health or social professional	242 (56.5)	556 (77.7)	525 (71.1)	<0.001
Not involved in care planning as much as the caregiver would like	187 (43.6)	345 (48.6)	324 (44.4)	NS
Caring experiences not valued enough by health and social services	129 (30.2)	324 (45.5)	243 (33.3)	<0.001
Not been able to take a break from caring	38 (8.9)	62 (8.8)	84 (11.3)	NS
Did not receive all the necessary equipment from health and social services	83 (20.0)	102 (14.7)	114 (16.0)	NS
Have not had enough help from health and social services with personal care of the patient	110 (26.6)	123 (18.2)	138 (19.5)	0.002
Have not had enough help from health and social services with household duties	103 (25.2)	167 (24.2)	163 (23.1)	NS
Have not had enough help from health and social services with health care	73 (18.0)	96 (14.4)	109 (15.6)	NS
Have not had enough help from health and social services with physical care	131 (32.1)	149 (21.8)	163 (23.6)	<0.001
Do not receive financial support from health and social services, but would like some	95 (22.5)	159 (23.2)	125 (17.6)	0.023
Had difficulty with obtaining financial support from health and social services	113 (27.2)	141 (20.7)	116 (16.8)	<0.001

**Table 4:** Frequencies of problems reported by LTNC (p<0.001)

	**MND**		**MS**		**PD**	
	**n**	**%**	**n**	**%**	**n**	**%**
**No problems**	42	12.1	32	5.5	41	7.2
**1 problem**	60	17.3	150	25.6	168	29.6
**2-3 problems**	98	28.3	180	30.7	165	29.0
**4-5 problems**	82	23.7	110	18.8	93	16.4
**6+ problems**	64	18.5	114	19.5	101	17.8

### Factors related to quality of life and strain

First, the individual associations between the total problem score and quality of life and carer strain were examined. PCS, MCS and CSI scores were significantly related to the number of problems reported (all p<0.001) (Table 5). Second, the individual associations between demographic and caregiving variables and related to quality of life and carer strain were examined. The PCS was significantly lower in PD (<0.001), the greater the age (p<0.001), longer duration of being a carer (p=0.03) and a higher number of hours caring per week (p<0.001). The MCS was significantly higher in MS (p<0.001), in women (p<0.001), with younger age (p=0.009), in those with a higher number of hours caring per week (p<0.001). The CSI score was significantly higher in MND (p<0.001), in women (p<0.001), in those with longer duration of being a carer (p=0.011) and a higher number of hours caring per week (p<0.001).

**Table 5:** Associations between the number of problems reported and demographic and caregiving variables

	**N problems reported**							
	**SF-12**					**CSI**		
		**PCS**		**MCS**				
	**N**	**Mean**	**95% CI**	**Mean**	**95% CI**	**N**	**Mean**	**95% CI**
**None**	102	51.06	48.98 – 53.13	48.13	46.20 - 50.06	100	11.10	9.96 – 12.5
**1**	349	49.75	48.64 – 50.85	48.93	47.87 -49.99	343	8.25	7.61 – 8.88
**2-3**	402	48.74	47.71 – 49.76	46.17	45.16 -47.19	389	11.61	11.00 – 12.22
**4-5**	245	47.13	45.70 – 48.56	41.48	40.13 -42.82	240	14.85	14.12 – 15.57
**6+**	243	44.18	42.58 - 45.76	38.26	36.84 – 39.68	227	17.11	16.37 – 17.85
**p**		<0.001		<0.001			<0.001	
	**LTNC**							
	**SF-12**					**CSI**		
		**PCS**		**MCS**				
	**N**	**Mean**	**95% CI**	**Mean**	**95% CI**	**N**	**Mean**	**95% CI**
**MND**	363	48.04	46.83 – 49.25	43.01	41.82 -44.21	362	14.69	14.01 – 15.38
**MS**	641	48.79	47.93 – 49.65	46.12	45.28 -46.96	633	11.44	10.93 – 11.95
**PD**	626	46.24	45.31 -47.16	44.02	43.16 – 44.89	571	11.89	11.36 – 12.41
**p**		<0.001		<0.001		<0.001		
	**Age**							
	**SF-12**					**CSI**		
		**PCS**		**MCS**				
	**N**	**Mean**	**95% CI**	**Mean**	**95% CI**	**N**	**Mean**	**95% CI**
**<55**	422	51.92	50.95 – 52.89	43.70	42.57 – 44.83	424	12.18	11.53 – 12.84
**55-63**	439	49.39	48.35 – 50.43	43.78	42.74 – 44.83	419	13.12	12.47 – 13.78
**64-71**	415	46.82	45.77 – 47.87	45.74	44.70 – 46.78	389	12.18	11.56 – 12.81
**72+**	354	41.35	40.12 – 42.58	45.45	44.35 – 46.56	334	11.80	11.10 – 12.50
**p**		<0.001		0.009		0.036		
	**Gender**							
		**SF-12**				**CSI**		
		**PCS**		**MCS**				
	**N**	**Mean**	**95% CI**	**Mean**	**95% CI**	**N**	**Mean**	**95% CI**
**Male**	689	48.20	47.37 – 49.04	46.41	45.58 – 47.23	681	11.33	10.84 – 11.82
**Female**	938	47.21	46.45 – 47.97	43.31	42.61 – 44.02	881	13.13	12.69 – 13.57
**p**		0.087		<0.001		<0.001		
	**Time since becoming a carer (years)**							
		**SF-12**				**CSI**		
		**PCS**		**MCS**				
	**N**	**Mean**	**95% CI**	**Mean**	**95% CI**	**N**	**Mean**	**95% CI**
**< 1**	107	49.63	47.40 – 51.85	44.26	42.15 – 46.38	115	11.70	10.51 -12.90
**1-2**	288	48.91	47.61 – 50.22	44.64	43.36 – 45.92	276	12.14	11.30 -12.97
**3-4**	268	47.41	45.97 – 48.84	44.43	43.00 - 45.85	259	11.78	10.96 – 12.60
**5-10**	466	48.05	47.05 – 49.06	44.80	43.78 - 45382	432	12.11	11.50 12.73
**> 10**	493	46.12	45.07 – 47.17	44.46	43.50 – 45.42	477	13.27	12.70 13.84
**p**		0.003		0.984		0.011		
	**Time spent caring (hours/week)**							
		**SF-12**				**CSI**		
		**PCS**		**MCS**				
	**N**	**Mean**	**95% CI**	**Mean**	**95% CI**	**N**	**Mean**	**95% CI**
**1-19**	540	49.83	48.92 – 50.74	47.85	47.00 – 48.70	525	8.09	7.65 – 8.23
**20-34**	274	47.47	46.12 - 48.81	44.16	42.94 45.37	279	12.15	11.50 – 12.79
**35-70**	346	47.33	46.11 – 48.55	42.42	41.19 – 43.65	333	14.60	13.97 – 15.24
**> 70**	429	44.74	43.57 45.91	42.39	41.28 – 43.50	388	16.77	16.17 17.37
**p**		<0.001		<0.001		<0.001		

Finally, the number of problems reported together with LTNC and demographic and caregiving variables as the covariates were entered into an ANCOVA with PCS, MCS and CSI scores as the outcome variables. All models were significant (p<0.001), showing that generally PCS and MCS decreased as a higher number of problems was reported and that CSI scores increased with a higher number of problems reported. The mean adjusted PCS, MCS and CSI scores by number of problems reported and the levels of significance for each variable in the model are presented in Table 6.

**Table 6:** Factors associated with carer quality of life and strain

	**SF-12**			**CSI**	
**Problem score**		**SF-12 PCS**	**SF-12 MCS**		
	**n**	**Mean (SE)**	**Mean (SE)**	**n**	**Mean (SE)**
0	99	51.1 (1.09)	48.3 (1.05)	98	11.3 (0.52)
1	333	48.6 (0.65)	48.0 (0.63)	327	10.1 (0.31)
2-3	391	48.2 (0.57)	46.2 (0.55)	378	12.0 (0.28)
4-5	243	46.9 (0.71)	42.2 (0.55)	237	13.9 (0.24)
6+	240	44.6 (0.72)	39.0 (0.69)	226	16.0 (0.35)
p		<0.001	<0.001		<0.001
**LTNC**	**n**	**Mean (SE)**	**Mean (SE)**	**n**	**Mean (SE)**
MND	297	48.3 (0.67)	44.1 (0.65)	301	14.1 (0.32)
MS	527	47.4 (0.64)	46.3 (0.62)	521	11.3 (0.31)
PD	482	47.9 (0.59)	43.8 (0.58)	444	12.4 (0.29)
p		0.634	0.003		<0.001
**Age (years)**	**n**	**Mean (SE)**	**Mean (SE)**	**n**	**Mean (SE)**
<55	357	51.9 (0.64)	42.8 (0.62)	365	13.3 (0.30)
55-63	364	50.2 (0.61)	42.9 (0.59)	348	13.7 (0.30)
64-71	315	46.7 (0.65)	45.9 (0.63)	299	12.4 (0.32)
72+	270	42.8 (0.71)	47.2 (0.69)	254	11.1 (0.35)
p		<0.001	<0.001		<0.001
**Gender**	**n**	**Mean (SE)**	**Mean (SE)**	**n**	**Mean (SE)**
Male	575	48.2 (0.53)	45.6 (0.51)	560	12.2 (0.26)
Female	731	47.6 (0.46)	43.9 (0.45)	706	13.0 (0.22)
p		0.357	0.006		0.010
**Time since becoming a carer (years)**	**n**	**Mean (SE)**	**Mean (SE)**	**n**	**Mean (SE)**
<1	92	48.6 (1.16)	45.7 (1.13)	95	11.5 (0.55)
1-2	236	48.6 (0.74)	44.2 (0.72)	230	12.5 (0.36)
3-4	205	47.3 (0.78)	44.5 (0.75)	202	12.6 (0.37)
5-10	375	48.3 (0.59)	44.8 (0.57)	361	13.0 (0.29)
>10	398	46.6 (0.63)	44.5 (0.61)	378	13.6 (0.31)
p		0.151	0.829		0.015
**Length of time spent caring/week (hours)**	**n**	**Mean (SE)**	**Mean (SE)**	**n**	**Mean (SE)**
1-19	451	49.3 (0.58)	47.0 (0.56)	442	8.90 (0.28)
20-34	221	47.2 (0.75)	45.1 (0.72)	224	11.8 (0.35)
35-70	293	48.2 (0.67)	43.1 (0.65)	287	14.2 (0.33)
>70	341	46.9 (0.64)	43.7 (0.62)	313	15.7 (0.32)
p		0.026	<0.001		<0.001
**Adjusted R squared**		0.13	0.17		0.42

## Discussion

The aim of this article was to examine the relationship between carer quality of life and strain and experiences of health and social care services. The findings show that quality of life is compromised compared with the general working age population and that carers in LTNC experience strain from their caregiving role. This is in line with previous findings [2,4,6-8]. Furthermore, differences were found in quality of life and strain between the carers of the three different LTNCs. Significant differences were found in the number of carers reporting problems with health and social services (6 out of 12 items) between the three LTNCs. The most commonly reported problem, reported by 69.3% overall, was not to have had any or enough opportunity to discuss the amount of caring with a health or social profession. Overall, there was no clear trend for carers of one of the LTNCs to report a more problems with individual items and there was no significant difference in terms of the total problem score. However, in individuals with one of the LTNCs, a trend was found with usually individuals with MND reporting fewer problems [29]. This seems to indicate that the problems reported by carers are less driven by the disease than by the problems reported by the patients.

The total problem score was however significantly associated with the PCS, MCS and CSI score, indicating that carers who reported more problems with health and social services had worse quality of life and higher strain. Previous studies have reported factors that impact on quality of life, for example duration of illness and the amount of time spent caring [4,7,10,11,30]. Therefore the significant relationship between carer well-being and experiences of services as measured by the total problem score was examined by including demographic and caring variables as covariates. The significant relationship persisted when LTNC and a range of demographic and caregiving covariates were included in the analysis. The association was particularly strong for carer strain, and less strong (but still significant) for quality of life. This is not unexpected as quality of life was measured by a generic health status questionnaire and carer strain by a more specific instrument.

Some limitations of the study need to be acknowledged. Overall, although a larger sample was achieved in this survey in comparison with previous carer studies [5,7,18,31], a carer responded for 37.4% of the patients contacted, and therefore caution is needed when interpreting the results. Taking this estimate as a response rate assumes that every patient has a caregiver, which is not necessarily the case, and investigating other means of recruiting caregivers may be important. Another limitation may be the recruitment through patient organisations, although there is no evidence to suggest that these carers are different from other carers. Finally, the results are based on a cross-sectional survey and therefore it is not possible to establish a causal link between quality of life and strain and the number of problems reported. However, the results do indicate that there is an association and therefore providing high quality support to carers of someone with a LTNC is important.

## Conclusions

This study set out to investigate the relationship between carer well-being and experiences of health and social care services in three long-term neurological conditions. The findings suggest that a considerable number of carers experience problems with health and social care. The number of problems reported is associated with carer quality of life and strain, even when other influencing factors (demographics, caregiving variables) are corrected for. This stresses the importance of health and social services appropriately supporting carers, and thus helping to minimise the burden caused by caring for someone with a LTNC. It is increasingly recognised that carers of people with long-term conditions play a crucial role in complementing the support given by health and social services. However, carers have their own needs and it may be that those with greater needs have less access to services to help them.

## Abbreviations

MS: Multiple sclerosis; MND: Motor neuron disease; PD: Parkinson’s disease; LTNC: Long-term neurological conditions; PCS: Physical component summary; MCS: Mental component summary; CSI: Carer strain index; ANOVA: Analysis of variance; ANCOVA: Analysis of covariance.

## Competing interests

The authors declared that they have no competing interests.

## Authors’ contributions

RF, CJ, DP and HD conceived and designed the study. All authors contributed to the development of the questionnaire. MP collected data. MP, RF, CJ, HD analysed data. MP wrote the article. All authors had access to the data in the study, and can take responsibility for the integrity of the data and the accuracy of the data analysis, and edited and approved the final version.
